# Case Report: A novel variant in fibrillin-2 identified in a congenital contractural arachnodactyly family with phenotypic heterogeneity

**DOI:** 10.3389/fmed.2026.1828971

**Published:** 2026-06-01

**Authors:** Nan-Miao Wang, Zhen-Bo Cheng, Xuan Yu, Ying-Nan Wang, Ze-Xuan Wang, Rui-Cheng Yao, Xin Jin, Jie-Yuan Jin

**Affiliations:** 1 School of Medicine, Shaoxing University, Shaoxing, China; 2 Department of Clinical Laboratory, Affiliated Hospital of Shaoxing University, Shaoxing, China; 3 Department of Clinical Laboratory, Hunan Provincial People’s Hospital, The First Affiliated Hospital of Hunan Normal University, Hunan Normal University, Changsha, China; 4 School of Life Sciences, Central South University, Changsha, China; 5 Department of Hand and Microsurgery, Xiangya Hospital, Central South University, Changsha, China

**Keywords:** arthrogryposis, congenital contractural arachnodactyly, crumpled ear, FBN2, phenotypic heterogeneity

## Abstract

**Background and objectives:**

Congenital contractural arachnodactyly (CCA) is a rare autosomal dominant connective tissue disorder, and *FBN2* is its only known causative gene. CCA is characterized by joint contractures, arachnodactyly, scoliosis, and crumpled ears. Due to its rarity, phenotypic diversity, heterogeneity, and clinical overlap with conditions such as Marfan syndrome (MFS), the diagnosis remains challenging, and genetic screening plays a critical role in facilitating accurate diagnosis. We recruited a CCA family with three patients across three generations and detected their genetic etiology.

**Case presentation:**

The proband exhibited the Marfanoid habitus with a height of 121 cm (> + 3 SD), a weight of 16 kg (−2 SD ∼−1 SD), arachnodactyly, and long bone overgrowth. He had joint contractures in the 2nd ∼ 5th fingers of bilateral hands and 2nd and 5th toes of the left foot. His mother and grandmother also presented arachnodactyly and arachnodactyly. They were confirmed to be affected with CCA.

**Results:**

A novel heterozygous missense variant in the exon 30 of *FBN2* (NM_001999.4: c.3916T > G, p.Y1306D) was identified by whole-exome sequencing. The variant was classified as “likely pathogenic” according to the American College of Medical Genetics and Genomics guidelines and standards. Bioinformatics predictions revealed that the variant altered the hydrophobicity, extended an intrinsically disordered protein region, disrupted a benzene ring structure on a β-sheet, and modified the surface charge of the fibrillin-2 partial region.

**Conclusion:**

We descripted a CCA family and identified a novel *FBN2* variant. Our findings extended the variant spectrum of *FBN2*, contributing to the genetic counseling and molecular diagnostics for CCA.

## 1 Introduction

Congenital contractural arachnodactyly (CCA, OMIM_121050) is a rare autosomal dominant connective tissue disorder characterized by joint contractures (predominantly involving interphalangeal, elbow, and knee joints), arachnodactyly, scoliosis, and crumpled ears. Additional secondary phenotypes include tall and slim stature, micrognathia, sternal deformities, muscle hypoplasia, and cardiovascular malformations (1, 2). Due to the rarity of CCA, combined with its phenotypic diversity, heterogeneity, and clinical overlap with conditions such as Marfan syndrome (MFS, OMIM_154700), the diagnosis is challenging (3). In fact, CCA was once regarded as a subtype of MFS. Accurate diagnosis is critical for prognosis and clinical management, making genetic screening an essential tool for confirming the disorder.

FBN2 is the only known causative gene for CCA, which is located at 5q23.3 and has 65 exons (4). It encodes a connective tissue protein, fibrillin-2, containing 2912 amino acids (5). Fibrillin-2 can be secreted into the extracellular matrix and participate in the assembly of microfibrils to maintain the structural integrity and flexibility of connective tissue. Fibrillin-2-containing microfibrils also regulate osteoblast maturation by modulating TGF-β bioavailability and calibrating TGF-β and BMP signaling (6). Since fibrillin-2 is widely expressed in human tissues, FBN2 variants result in a broad spectrum of clinical manifestations, including arachnodactyly, macular degeneration, skeletal malformations, scoliosis, and arterial/valvular diseases such as thoracic aortic disorder and mitral valve prolapse (5, 7, 8). While the most well-known disease caused by FBN2 variant remains CCA. However, the detection rate of FBN2 variants in CCA remains below 75%, suggesting either locus heterogeneity or clinical overdiagnosis of CCA (3). Biallelic FBN2 variants can cause the more severe CCA, showing that the disease exhibits incomplete dominance (9). In addition, large-scale forward genetic screening in mouse showed that Fbn2^*T2547A*/*T2547A*^ mice presented the shorter, narrowed tracheas and digit fusions, and these phenotypes have not yet been identified in human clinical cases (10). These facts demonstrate the importance of reporting disorders associated with FBN2 variants. More human genetic studies and reports would help us gain a deeper understanding of the genotype-phenotype correlations of FBN2, as well as the new genetic pathogenic factors of CCA.

In this study, we described a CCA family with three patients across three generations and identified a novel *FBN2* variant (NM_001999.4: c.3916T > G, p.Y1306D) in this family. Our findings extended the known repertoire of *FBN2* variants, contributing to the genetic counseling and molecular diagnostics for CCA.

## 2 Materials and methods

### 2.1 Subjects

**FIGURE 1:**
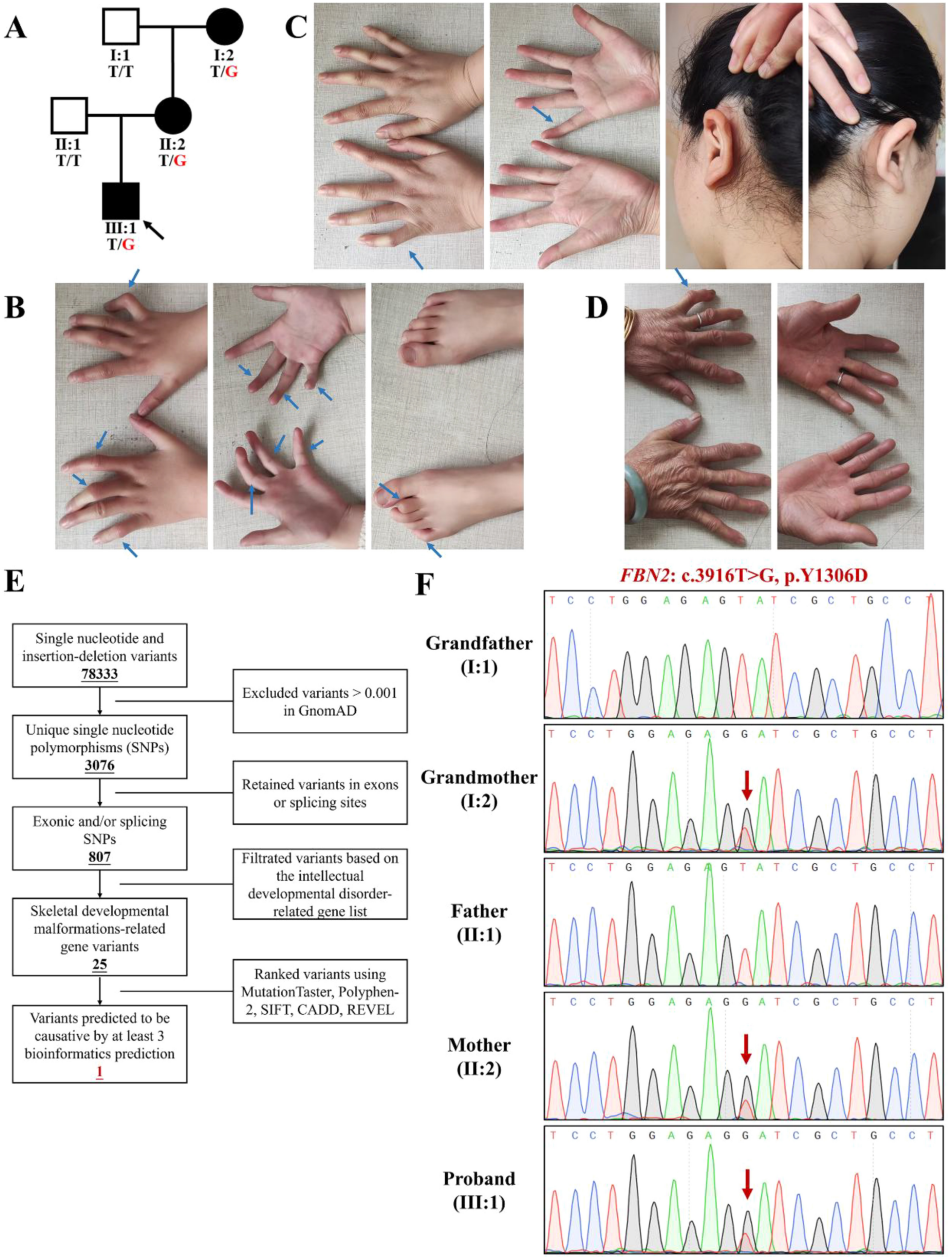
The symptoms and Sanger sequencing results of the family. **(A)** Family pedigree of the proband. The pedigree of this family. Black circles/squares are affected, white circles/squares are unaffected, the arrow indicates the proband, and red text represents the variant. **(B–D)** The phenotypes of the proband **(B)**, II:2 **(C)**, and I:2 **(D)**. **(E)** The strategy of genetic screening in this study. **(F)** The proband (III:1), II:2, and I:2 harbored the FBN2 variant (c.3916T > G, p.Y1306D), while II:1 and I:1 did not. Red arrows indicate variants sites.

This study obtained the consent of the subjects and received approval from the ethics committee of School of Medicine, Shaoxing University (2024C073). A CCA family with three affected individuals across three generations was recruited (Figure 1A and Table 1). The proband was a minor, and written informed consents were obtained from the guardians of the proband and other participants for their involvement in this study and the publication of clinical details/images. Genomic DNAs were extracted from the peripheral blood of all participants using the DNeasy Blood & Tissue Kit (Qiagen, Valencia, USA).

**TABLE 1:** Clinical details of the patients in this CCA family.

Patient		Grandmother (I:2)	Mother (II:2)	Proband (III:3)
Gender		Female	Female	Male
Age (year)		57	33	4
Height (cm)		158 (normal)	162 (normal)	121 (> + 3 SD)
Weight (kg)		64 (normal)	61 (normal)	16 (−2 SD ∼−1 SD)
Long bone overgrowth (score)		− (–)	− (–)	+ (3)
Arachnodactyly (score)		+ (3)	+ (3)	+ (3)
Joint contracture (score)	Hand	The 5th finger of the left hand (3)	Bilateral 5th fingers (3)	The 2nd ∼ 5th fingers of bilateral hands
	Foot	−	−	The 2nd and 5th toes of the left foot (3)
Crumpled ear (score)		− (−)	+ (3)	− (−)
Other (score)		Hypertension (−)	− (−)	− (−)
Total score*		6	9	9

*A clinical score of ≥ 7 is suggestive for CCA, and a score of ≥ 11 makes the diagnosis of CCA likely. +, affected; −, unaffected. SD, standard deviation; −3 SD, 3% population; −2 SD, 10% population; −1 SD, 25% population; SD, 50% population; +1 SD, 75% population; +2 SD, 90% population; +3 SD, 97% population.

### 2.2 Whole-exome sequencing and variant analysis

Whole-exome sequencing (WES), encompassing exome capture, high-throughput sequencing, and routine annotation, was conducted by Berry Genomics Company Limited (Beijing, China) (details seen Supplementary materials). Our target variants in this study were restricted to exons and/or splicing sites. And in these variants, if someone had a detection rate ≥ 0.001 in GnomAD,^1^ it was eliminated. MutationTaster,^2^ Polyphen-2,^3^ SIFT,^4^ CADD,^5^ and REVEL^6^ were used to predict the pathogenicity of these variants (11). A missense variant was retained when at least three prediction tools determined it as disease-causing, while null variants were retained by default. For these selected variants, their phenotypes and inheritance patterns were annotated by OMIM,^7^ and adhered to the standards and guidelines of the American College of Medical Genetics and Genomics (ACMG), their pathogenicity classifications were determined manually (12). Each piece of supportive pathogenic evidence is assigned +1 point, moderate evidence is assigned +2, strong evidence is +4, and very strong evidence is +8. A final score between 6 and 9 points is classified as likely pathogenic, and a score of 10 points or above is classified as pathogenic (13). Pathogenic or likely pathogenic variants were designated as candidate variants.

### 2.3 Sanger sequencing

The candidate variants were validated through Sanger sequencing. Variant sites and their flanking sequences were acquired from the NCBI database.^8^ The *FBN2* primer pair (F: 5′-CCAGATATTACCAAGTGGAACCAG-3′, R: 5′-ATGGAAGCCATGAAGCCATC-3′) was designed by IDT.^9^ Sanger sequencing was performed by Sangon Biotech Company Limited (Shanghai, China). Sanger sequencing was performed using the dideoxy chain termination method. Briefly, the DNA template was extracted and purified. Sequencing PCR was carried out with sequencing primers, dNTPs, fluorescently labeled ddNTPs and Taq DNA polymerase. The amplicons were purified and denatured, followed by capillary electrophoresis to separate DNA fragments of different lengths. Fluorescence signals were detected by laser, and electropherograms were analyzed to obtain the target gene sequence.

### 2.4 Function prediction

FBN2 protein sequences from multiple species, obtained from NCBI,^10^ were used for conservation analysis. The hydrophobicity of the wild-type and mutant FBN2 proteins was predicted by Expasy^11^ and visualized by GraphPad Prism 10. The intrinsically disordered protein regions (IDRs) of the wild-type and mutant FBN2 proteins were predicted by PONDR^12^ and visualized by GraphPad Prism 10. The three-dimensional protein model of the wild-type FBN2 was established by the AlphaFold 3 database,^13^ and the mutant FBN2 model was made by PyMol.

## 3 Results

### 3.1 Case description

The proband (III:1; Figure 1A) was a 4.5-year-old boy, and his mother (II:2) presented him to our hospital to consult whether he was affected with MFS. He exhibited the Marfanoid habitus, measuring 121 cm in height (> + 3 SD) and weighing approximately 16 kg (−2 SD ∼−1 SD), with arachnodactyly and long bone overgrowth (the arm span of approximately 126 cm and the lower limb length of approximately 67 cm) (Table 1). Given that he had joint contractures in the 2nd ∼ 5th fingers of bilateral hands and 2nd and 5th toes of the left foot (Figure 1B), and the absence of other systemic symptoms, a diagnosis of CCA was prioritized over MFS. Moreover, in accordance with the clinical scoring system for CCA, the proband scored 9 points, indicating a diagnosis of CCA (14).

Tracing his family history, both his mother (II:2) and grandmother (I:2) also had similar phenotypes (Table 1). II:2 was 33 years old and with a normal stature. She exhibited arachnodactyly, arthrogryposis of bilateral 5th fingers, and crumpled ears, and her CCA score was 9 points (Figure 1C). I:2 was a 57-year-old woman with average build. She had arachnodactyly, and only the 5th finger of the left hand was involved in joint contracture (Figure 1D). Her score was only 6 (< 7 points), failing to meet the diagnostic criteria for CCA. In addition, she was diagnosed with hypertension at 46 years of age. The father and grandfather of the proband were unaffected.

### 3.2 Genetic analysis

For determining their diagnosis, we performed WES on the proband (No. PRJCA043749), which generated 10.6 GB data covering 99.53% target region (98.31% achieving a sequencing depth of ≥ 10×). According to strategy as Figure 1E, a novel heterozygous variant in the exon 30 of *FBN2* (NM_001999.4: c.3916T > G, p.Y1306D) was identified (Table 2). Sanger sequencing confirmed co-segregation of this variant with the phenotype: all three affected individuals (I:2, II:2, III:1) carried the variant, while unaffected family members did not (Figure 1F).

**TABLE 2:** The information and pathogenicity classification of the *FBN2* variant identified in the proband.

Gene	Variant	Inheritance	Pathogenicity prediction*	GnomAD	OMIM clinical phenotype	American College of Medical Genetics classification
*FBN2*	NM_001999.4: c.3916T > G, p.Y1306D	From mother	MutationTaster: D Polyphen-2: D SIFT: D CADD:28.5 REVEL: 0.960	Null	AD, contractural arachnodactyly, congenital; AD, macular degeneration, early-onset.	Likely pathogenic (PM1, PM2, PP1, PP3_moderate) Point: 7 (2 + 2 + 1 + 2)

*“D” means disease-causing, and the ACMG criteria is PP3; the ACMG criteria is PP3 when 25.3 ≤ CADD score < 28.1, and is PP3_ moderate when CADD score ≥ 28.1; the ACMG criteria is PP3 when 0.644 ≤ REVEL score < 0.773, is PP3_ moderate when 0.773 ≤ REVEL score < 0.932, and is PP3_strong when REVEL score ≥ 0.932 (based on the paper PMID:36413997). We compromise on choosing PP3_ moderate. AD, autosomal dominant.

The pathogenicity classification of ACMG showed that this variant was likely pathogenic, supported by the following criteria: (1) This variant was positioned on a critical functional domain, the EGF-like domain 20 (1,283th ∼ 1,324th amino acids; PM1); (2) This variant was not recorded in GnomAD database, which is the world’s largest genetic database for normal populations (PM2); (3) The genotype (c.3916T > G) and the phenotype (CCA) conformed to the co-segregation in this pedigree (PP1); (4) MutationTaster, Polyphen-2, SIFT, CADD, and REVEL all predicted this variant to be causative, and all the scores were very high (PP3_ moderate; Table 2).

**FIGURE 2:**
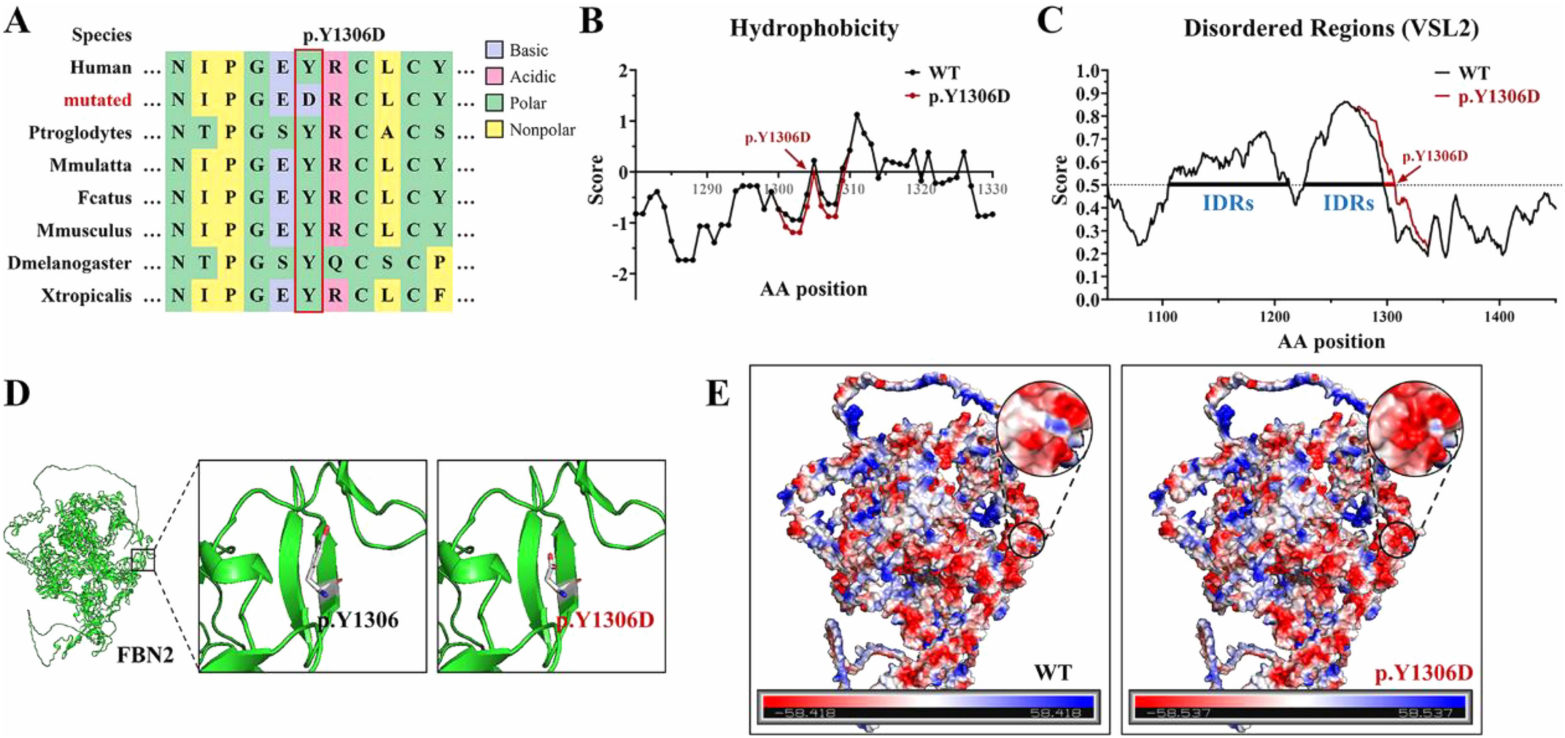
The function prediction of the mutant fibrillin-2. **(A)** Conservation analysis of the mutant amino acid site of fibrillin-2. **(B)** The hydrophobic prediction of fibrillin-2 fragment with wild type (black) and p.Y1306D (red). **(C)** The intrinsically disordered protein regions (IDRs) prediction of fibrillin-2 fragment with wild type (black) and p.Y1306D (red). **(D)** Protein modeling of fibrillin-2 with wild type and p.Y1306D. **(E)** The surface charge prediction of fibrillin-2 with wild type and p.Y1306D.

Amino acid sequence alignment analysis revealed that the variant site exhibited high evolutionary conservation (Figure 2A). Upon further analysis of the impact of the variant p.Y1306D on the FBN2 protein, we found that this variant slightly increased the hydrophilicity the surrounding area of the variant and facilitated the intrinsically disordered of FBN2, resulting in the extension of one IDR (Figures 2B,C). Protein modeling results showed that the variant occurred on a β-sheet and that the substitution of tyrosine with aspartic acid lost a benzene ring structure and changed the surface charge of FBN2 protein (Figures 2D,E). Collectively, all these results suggested that the *FBN2* variant (c.3916T > G, p.Y1306D) was the genetic etiology of this family, and the diagnosis of these patients was CCA, although the score of I:2 did not meet the score of the diagnostic criteria for CCA.

## 4 Discussion

CCA is a connective tissue disease with diverse phenotypes, whose core symptoms include arachnodactyly, camptodactyly, scoliosis, and crumpled ear. Most patients (including our proband) initially present to orthopedic clinics due to joint contractures (particularly interphalangeal joints) and are often misdiagnosed as having distal arthrogryposis in the early stage of diagnosis (15, 16). Except distal arthrogryposis, CCA is often confused with MFS, especially in patients with Marfanoid features (1). However, the fatality rates of these two diseases are completely different. Additionally, part CCA phenotypes overlap with type VI collagenopathies, Ullrich congenital muscular dystrophy, and Loeys–Dietz syndrome (3). Thus, molecular diagnosis is crucial for the definitive CCA confirmation. The diagnostic criteria of CCA did not include the genetic evidence. As the main causes of CCA, the identification of *FBN2* variants should be incorporated into the diagnostic workup, as is the practice for familial hypercholesterolemia. In this study, upon identifying the *FBN2* variant (c.3916T > G, p.Y1306D), we confirmed the family’s diagnosis of CCA, despite their classic clinical manifestations. Since the proband still retained satisfactory manual function of the right hand for writing and using chopsticks, no surgical or other subsequent interventions had been performed. The proband’s mother initially presented the child to our hospital mainly due to concern about a potential diagnosis of MFS. Although MFS was ultimately excluded, given that a few *FBN2* carriers developed life-threatening conditions such as aortic dissection, we recommend that these patients undergo regular physical examinations and annual clinical follow-up.

CCA exhibits the phenotypic heterogeneity, which was evident among our patients. The three patients from one family harbored the same *FBN2* variant but displayed variable symptoms: the proband had the most severe phenotype, with contractures involving multiple finger and toe joints; his grandmother had milder arthrogryposis; and only his mother had crumpled ears. Jensen et al. (17) reported that genetic background exerts a substantial influence on disease pathogenesis and phenotypic manifestations (17). In our case, the phenotypic heterogeneity in the three generations of grandparents and grandchildren is attributed to differences in their genetic backgrounds, and cryptic minor-effect genes are transmitted and accumulated across generations, resulting in the proband exhibiting the most severe phenotype. Verification of this hypothesis requires corroboration through analysis of large sample sizes. It should be noted that owing to the lack of long-term follow-up, our clinical evaluation of the patients is limited to the current cross-sectional observation. Furthermore, given that the patients are at different life stages, a direct comparison of phenotypic severity between them is methodologically inappropriate.

*FBN1* and *FBN2* encode fibrillins, and their variants caused MFS and CCA, respectively (18). Fibrillins are the major components of extracellular microfibers and have widespread distribution in both elastic and nonelastic connective tissues throughout the body (2). Zhang et al. (19) suggested that the expression of fibrillin-2 directs the assembly of elastic fibers during early embryogenesis, while fibrillin-1 provides the major structural (such as load bearing) function of the microfibrils (20). Except function, their tissue distribution also differs. Fibrillin-2 is highly expressed in the mesenchymal tissue of the early embryo, particularly in the tendon and bone development areas of the limbs. In adulthood, fibrillin-2 is mainly concentrated in tendons and ligaments (21). While the tissue distribution of fibrillin-1 is more extensive, majorly in cardiovascular system, bone and arthrosis, and ocular tissue. Fibrillin-1 defects can result in more diverse phenotypes, including life-threatening aortic dissection (22). Our patients did not carry any *FBN1* causative variant, and we assessed that their risk of death due to CCA is relatively low, although the grandmother had hypertension (aortic dissection closely associated with hypertension). However, regular physical examinations remain necessary.

**FIGURE 3:**
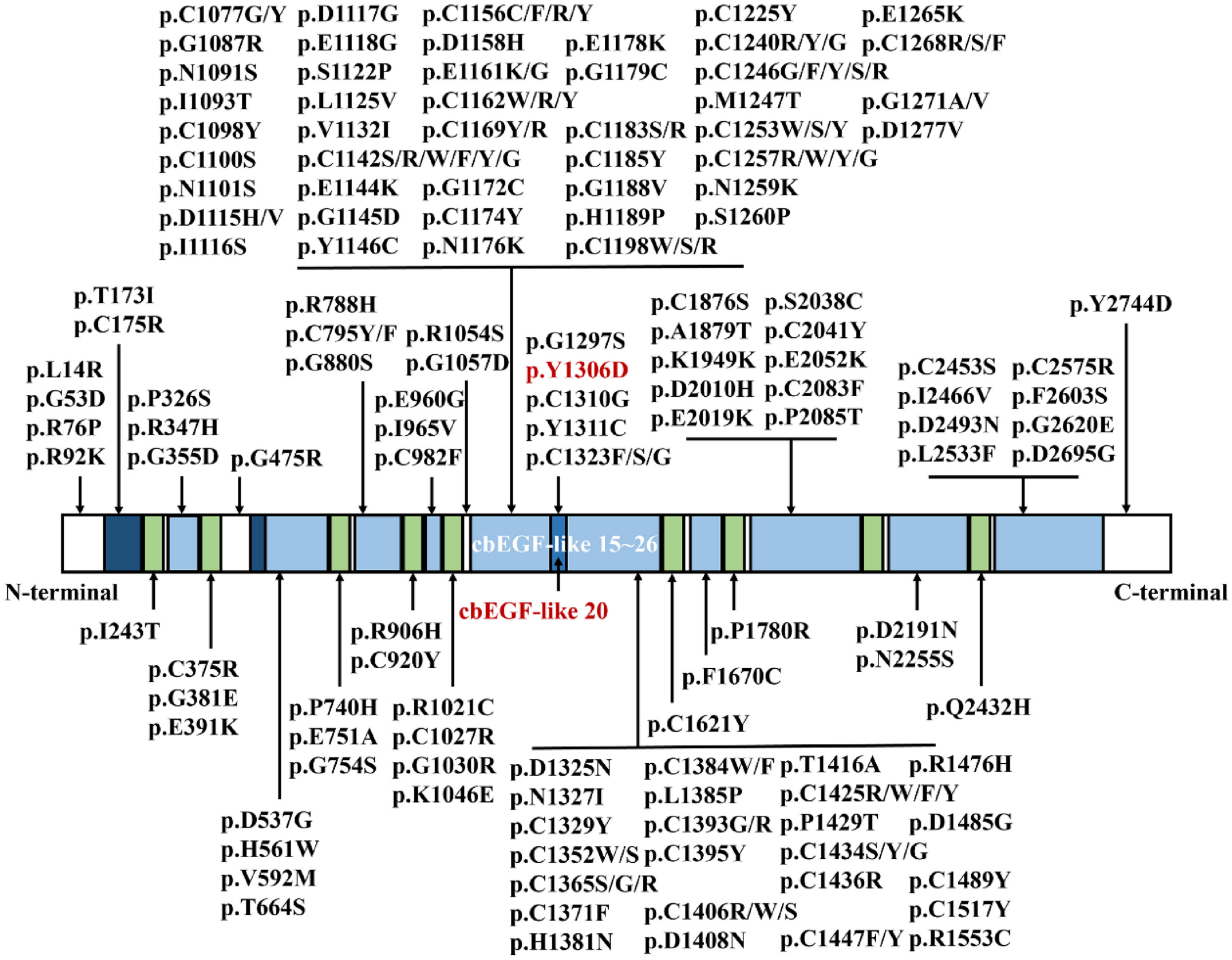
Summary of *FBN2* variants. *FBN2* variants reported in databases, literatures and this report. The boxes constitute the structure of fibrillin-2. Blue boxes represent EGF-like domains, green boxes represent TGF-β binding domains, and light blue boxes represent calcium-binding EGF-like domains. Our variant was positioned in calcium-binding EGF-like domain 20. Arrows and texts represent the variants in *FBN2*, in which the red fonts represent the variant in this study.

Fibrillin-2 mainly contains a N-terminal region, 47 EGF-like domains (43 calcium-binding domains and four non-calcium-binding domains), nine TGF-β binding domains, and a C-terminal unique domain (10, 23). To date, at least 181 missense variants in *FBN2* have been reported, and calcium-binding EGF-like (cbEGF-like) domains 15 ∼ 26 are in the mutational hot spot (Figure 3) (4, 9, 15, 16, 18, 24–30). Our variant p.Y1306D localizes to the cbEGF-like domain 20 (1,283th ∼ 1,324th amino acids) and was predicted to alter the hydrophobicity, IDR, benzene ring structure, and surface charge of the local region. It may impact functions of fibrillin-2, for example the interaction with other proteins, which requires further validation. Consistent with our findings, other missense variants in this domain (such as p.C1323G and p.C1323S) were also associated with CCA, while the variant p.G1297S was linked to thoracic aortic disorder (31). *In vitro* assays, point mutant mouse models, and single-cell sequencing can facilitate the elucidation of the pathogenic mechanisms underlying *FBN2* variants p.Y1306D, as well as the exploration of the origins of the associated phenotypic heterogeneity.

Overall, this case showed that the novel *FBN2* variant (c.3916T > G, p.Y1306D) was pathogenic and emphasized the important of genetic screening in CCA diagnosis, especially the patients with Marfanoid habitus.

## 5 Conclusion

In this study, we described a case where three generations carried the same *FBN2* variant but exhibited variable phenotypes, highlighting the phenotypic heterogeneity of CCA. We identified a novel variant (NM_001999.4: c.3916T > G, p.Y1306D) in the exon 30 of *FBN2* in a CCA family, expanding the genetic spectrum of CCA and emphasizing the value of *FBN2* screening in CCA diagnosis. We compiled known missense variants of *FBN2*, contributing to the genetic counseling and molecular diagnostics for CCA.

## Data Availability

The datasets presented in this study can be found in online repositories. The names of the repository/repositories and accession number(s) can be found below: https://www.cncb.ac.cn/, PRJCA043749.

## Footnotes

1
http://gnomad.broadinstitule.org

2
http://www.mutationtaster.org/

3
http://genetics.bwh.harvard.edu/pph2/

4
http://provean.jcvi.org/index.php

5
https://cadd.gs.washington.edu/snv

6
https://sites.google.com/site/revelgenomics/

7
https://www.omim.org

8
https://www.ncbi.nlm.nih.gov/gene

9
https://sg.idtdna.com/Primerquest/Home/Index

10
https://www.ncbi.nlm.nih.gov/protein/?term=FBN2

11
https://web.expasy.org/protscale/

12
https://www.pondr.com/

13
https://alphafoldserver.com/
