# Supplementary material for: Case Report: A novel variant in fibrillin-2 identified in a congenital contractural arachnodactyly family with phenotypic heterogeneity
Source: Front Med (Lausanne). 2026 Jun 1;13:1828971. doi: 10.3389/fmed.2026.1828971 (PMC13265295; doi:10.3389/fmed.2026.1828971)
Supplement: Supplementary file 1 [file Data_Sheet_1.pdf]

CARE case report guidelines

CARE Checklist of information to include when writing a case report

(cc) BY-NC-ND

✓

Topic	Item	Checklist item description	Reported on Line
Title	1	The diagnosis or intervention of primary focus followed by the words "case report"	line 1-2
Key Words	2	2 to 5 key words that identify diagnoses or interventions in this case report, including "case report"	line 43-44
Abstract (no references)	3a	Introduction: What is unique about this case and what does it add to the scientific literature?	line 32-41
	3b	Main symptoms and/or important clinical findings	line 27-31
	3c	The main diagnoses, therapeutic interventions, and outcomes	line 32-37
	3d	Conclusion—What is the main "take-away" lesson(s) from this case?	line 40-41
Introduction	4	One or two paragraphs summarizing why this case is unique (**may include references**)	line 71-74
Patient Information	5a	De-identified patient specific information.	line 77-83
	5b	Primary concerns and symptoms of the patient.	line 119-124
	5c	Medical, family, and psycho-social history including relevant genetic information	line 125-145
	5d	Relevant past interventions with outcomes	line 125-131
Clinical Findings	6	Describe significant physical examination (PE) and important clinical findings.	line 119-131
Timeline	7	Historical and current information from this episode of care organized as a timeline	line 125-131
Diagnostic Assessment	8a	Diagnostic testing (such as PE, laboratory testing, imaging, surveys).	line 119-131
	8b	Diagnostic challenges (such as access to testing, financial, or cultural)	line 138-154
	8c	Diagnosis (including other diagnoses considered)	line 133-154
	8d	Prognosis (such as staging in oncology) where applicable	line 119-124
Therapeutic Intervention	9a	Types of therapeutic intervention (such as pharmacologic, surgical, preventive, self-care)	line 133-137
	9b	Administration of therapeutic intervention (such as dosage, strength, duration)	line 133-137
	9c	Changes in therapeutic intervention (with rationale)	line 138-145
Follow-up and Outcomes	10a	Clinician and patient-assessed outcomes (if available)	line 138-154
	10b	Important follow-up diagnostic and other test results	line 146-154
	10c	Intervention adherence and tolerability (How was this assessed?)	line 138-154
	10d	Adverse and unanticipated events	line 146-154
Discussion	11a	A scientific discussion of the strengths AND limitations associated with this case report	line 197-198
	11b	Discussion of the relevant medical literature **with references**.	line 191-201
	11c	The scientific rationale for any conclusions (including assessment of possible causes)	line 138-145
	11d	The primary "take-away" lessons of this case report (without references) in a one paragraph conclusion	line 203-208
Patient Perspective	12	The patient should share their perspective in one to two paragraphs on the treatment(s) they received	line 165-168
Informed Consent	13	Did the patient give informed consent? Please provide if requested	Yes ☑ No ☐
