# Supplementary material for: Case Report: A novel variant in fibrillin-2 identified in a congenital contractural arachnodactyly family with phenotypic heterogeneity
Source: Front Med (Lausanne). 2026 Jun 1;13:1828971. doi: 10.3389/fmed.2026.1828971 (PMC13265295; doi:10.3389/fmed.2026.1828971)
Supplement: Supplementary file 2 [file Data_Sheet_2.pdf]

Whole Exome Sequencing

全外显子组测序方法

We took a Trio-WES strategy to identify the causal variants from the family trios. In brief, genomic DNA was extracted, hybridized and enriched.

采用 Trio-WES 策略进行家系病因变异识别。简要概括主要包括基因组 DNA 提取、杂交和富集。

Genomic DNA Extraction

DNA 提取

1μg genomic DNA was extracted from 200μL peripheral blood, using a Qiagen DNA Blood Midi/Mini kit (Qiagen GmbH, Hilden, Germany) following the manufacturer's protocol.

取 200 μl 外周血，按照操作说明书使用 Qiagen 试剂盒（Qiagen DNA Blood Midi/Mini kit (Qiagen GmbH, Hilden, Germany)）进行 DNA 提取，获得 DNA 样本（浓度≥3ng/μl）。

Library Preparation And Sequencing

文库准备和测序

50 nanogram DNA was interrupted to 200bp around by fragmentation enzymes. The DNA fragments were then end repaired, and the 3'end was added one A base. Secondly, the DNA fragments were ligated with barcoded sequencing adaptors, and fragments about 320bp were collected by XP beads. The DNA fragments were hybridized and captured by Berry's NanoWES Human Exome V2.0(Berry Genomics,Beijing,China) according to the manufacturer's Protocol. The hybrid products were eluted and collected, and then subjected to PCR amplification and the purification. The libraries were quantified by qPCR and size

distribution were determined using an Agilent Bioanalyzer 2100 (Agilent Technologies, Santa Clara, CA, USA).

取 50ng DNA 样本，利用片段化酶将 DNA 打断为 200 bp 左右的片段。将 DNA 片段进行末端修复，3’末端进行加碱基 A 处理。然后将 DNA 片段连接接头标签序列，用 AMPure XP 磁珠对形成的大小约 320 bp 的片段进行富集。使用贝瑞人类外显子 V2 探针（Berry’s NanoWES Human Exome V2.0(Berry Genomics,Beijing,China)）对连接后 DNA 片段进行杂交和捕获，将捕获 DNA 片段进行 PCR 扩增和纯化。使用 Qpcr 方法进行文库定量，使用安捷伦 Bioanalyzer 2100 (Agilent Technologies, Santa Clara, CA, USA)进行文库片段大小测定分析。

Finally, Novaseq6000 platform (Illumina, San Diego, USA), with 150 bp pair-end sequencing mode, was used for sequencing the genomic DNA of the family. Raw image files were processed using CASAVA v1.82 for base calling and generating raw data.

最后，使用 Novaseq6000 平台（Illumina, San Diego, USA）150bp 双端测序程序进行家系基因组 DNA 测序。利用 CASAVA v1.82 软件将测序原始图像文件进行碱基序列转化并生成原始 fastq 数据。

Data Analysis

The sequencing reads were aligned to the human reference genome (hg38/GRCh38) using Burrows–Wheeler Aligner tool and PCR duplicates were removed by using Picard v1.57 (http://picard.sourceforge.net/). Verita Trekker® Variants Detection System by Berry Genomics and the third-party software GATK (https://software.broadinstitute.org/gatk/) were employed for variant calling. Variant annotation and interpretation were conducted by ANNOVAR (Wang, et al, 2010) and the Enliven® Variants Annotation Interpretation System authorized by Berry Genomics. Annotation databases mainly included:

使用 BWA 软件将测序读取序列比对到人类参考基因组(hg38/GRCh38)上，用 Picard v1.57 软件(http://picard.sourceforge.net/)去除比对后的 PCR 扩增产生的重复序列。使用贝瑞基因 Verita Trekker®变异检测系统和第三代 GATK 软件 (https://software.broadinstitute.org/gatk/)进行变异读取。用贝瑞基因 Enliven® 变异注释解读系统和 ANNOVAR (Wang, et al, 2010)软件对读取变异进行注释和解读。

i) human population databases, such as gnomAD (http://gnomad.broadinstitute.org/), the 1000 Genome Project (http://browser.1000genomes.org), Berrybig data population database, dbSNP (http://www.ncbi.nlm.nih.gov/snp) etc;

i)人类人群数据库，如 gnomAD (http://gnomad.broadinstitute.org/)，千人基因组项目 (http://browser.1000genomes.org)，贝瑞人群大数据数据库，dbSNP (http://www.ncbi.nlm.nih.gov/snp)等。

ii) in silico prediction algorithms, such as SIFT (http://sift.jcvi.org), FATHMM (http://fathmm.biocompute.org.uk), Mutation Assessor(http://mutationassessor.org), CADD (http://cadd.gs.washington.edu), SPIDEX(Xiong et al, Science 2015), etc;

ii)计算机预测，如 SIFT (http://sift.jcvi.org), FATHMM (http://fathmm.biocompute.org.uk), Mutation Assessor(http://mutationassessor.org), CADD (http://cadd.gs.washington.edu), SPIDEX(Xiong et al, Science 2015)等。

iii) disease and phenotype databases, such as OMIM (http://www.omim.org), ClinVar (http://www.ncbi.nlm.nih.gov/clinvar), HGMD (http://www.hgmd.org),HPO (https://hpo.jax.org/app/) etc.

iii)疾病和表型数据库，如 OMIM (http://www.omim.org), ClinVar (http://www.ncbi.nlm.nih.gov/clinvar), HGMD (http://www.hgmd.org),HPO (https://hpo.jax.org/app/)等

The variants were classified to five categories --”pathogenic”, ”likely pathogenic”, ”uncertain significance”, ”likely benign” and ”benign”--according to the American College of Medical Genetics and Genomics (ACMG) guidelines for interpretation of genetic variants (Richards et al., 2015).Variants with minor allele frequencies (MAF) <1% in exonic region or with splicing impact were taken for deep interpretation considering ACMG category, evidence of pathogenicity, and clinical synopsis and inheritance model of the associated disease.

基于美国医学遗传和基因组学会（ACMG）的遗传变异解释指南(Richards et al., 2015)将变异分为 5 类：致病、疑似致病、意义不明确、疑似良性和良性。选取次等位基因频率（MAF）≤0.01 的变异，进一步结合 ACMG 分级、致病性证据、关联疾病临床表征和遗传模式进行解读。

For trio-analysis, potential monogenetic inheritance patterns including de novo, autosomal recessive, autosomal dominant, X-linked recessive inheritance, mitochondrial, and, where possible, imprinted gene variation were analyzed. Full penetrance was assumed for the potentially causal variants and variants that were found in the parents or were recorded in any of the above mentioned databases or in our in-house control exomes were excluded as etiology. Once a variant was considered as the etiology of a recessive disorder, manually inspection for coverage and additional variants of the entire coding domain was undertaken using Integrated Genomics Viewer.

对于家系三人分析，结合潜在的单基因遗传模式包括新发、常染色体隐性、常染色体显性、X 染色体连锁隐性、线粒体以及其他可能的印记基因，对变异进行分析。将假设具有完全外显率且在亲本中、记录在上述任何数据库或贝瑞内部对照外显子组中的发现的潜在病因变异排除。一旦变异被认为是隐性疾病的病因，会使用 IGV 软件手动检查整个编码域的覆盖率和其他变异。

Reference

1、Wang K, Li M, Hakonarson H. ANNOVAR: Functional annotation of genetic variants from next-generation sequencing data Nucleic Acids Research, 38:e164, 2010.

2、Hui Y. Xiong, Babak Alipanahi, Leo J. Lee, et al. The human splicing code reveals new insights into the genetic determinants of disease[J]. Science, 2015, 347(6218):1254806.

3、Richards S, Aziz N, Bale S, et al. Standards and Guidelines for the Interpretation of Sequence Variants: A Joint Consensus Recommendation of the American College of Medical Genetics and Genomics and the Association for Molecular Pathology[J]. Genetics in Medicine Official Journal of the American College of Medical Genetics, 2015, 17(5):405-424.
